# 1D-CNN-based audio tampering detection using ENF signals

**DOI:** 10.1038/s41598-024-60813-0

**Published:** 2024-05-16

**Authors:** Haifeng Zhao, Yanming Ye, Xingfa Shen, Lili Liu

**Affiliations:** The School of Computer Science and Technology, Hangzhou Dianzi University, Hangzhou, 310018 China

**Keywords:** Computer science, Information technology

## Abstract

The extensive adoption of digital audio recording has revolutionized its application in digital forensics, particularly in civil litigation and criminal prosecution. Electric network frequency (ENF) has emerged as a reliable technique in the field of audio forensics. However, the absence of comprehensive ENF reference datasets limits current ENF-based methods. To address this, this study introduces ATD, a blind audio forensics framework based on a one-dimensional convolutional neural network (1D-CNN) model. ATD can identify phase mutations and waveform discontinuities within the tampered ENF signal, without relying on an ENF reference database. To enhance feature extraction, the framework incorporates characteristics of the fundamental harmonics of ENF signals. In addition, a denoising method termed ENF noise reduction (ENR) based on the variational mode decomposition (VMD) and robust filtering algorithm (RFA) is proposed to reduce the impact of external noise on embedded electric network frequency signals. This study investigates three distinct types of audio tampering—deletion, insertion, and replacement—culminating in the design of binary-class tampering detection scenarios and four-class tampering detection scenarios tailored to these tampering types. ATD achieves a tampering detection accuracy of over 93% in the four-class scenario and exceeds 96% in the binary-class scenario. The effectiveness, efficiency, adaptability, and robustness of ATD in the two and four classification scenarios have been confirmed by extensive experiments.

## Introduction

In the era of the Internet of Things (IoT), security and privacy protection are the cornerstones of building consumer trust. As various smart consumer devices increasingly infiltrate our daily lives, they frequently collect and process personal information, including audio data. However, the security and integrity of audio data are also facing challenges, especially with the advancement of tampering techniques. Audio tampering can take many forms, ranging from simple clipping to intentional content modification using advanced software. In an IoT environment, if consumer devices or communication links are not secure enough, attackers could hijack these devices and tamper with audio data, whether stored or in transit. Against this backdrop, detecting audio data tampering in the IoT environment has become the key issue to address this tampering risk.

In recent years, the significance of electric network frequency (ENF) in identifying audio manipulation has been well-established. ENF, a dynamic signal generated by the electric network at either 50 or 60 Hz, displays slight variations in different temporal and spatial locations^1^. These inherent fluctuations in the ENF signal can be extracted from recorded audio and serve as a natural time stamp. This process holds promise for authenticating audio recordings, especially when recording devices capture the ENF signal within the magnetic field of the electric network. Analyzing the extracted ENF signal provides a means to determine the veracity of the audio.

The development of an ENF-based method for identifying audio tampering is attributed to Dr. Grigoras in 2005^2^. Current ENF-based algorithms for tampering identification generally rely on cross-referencing the electric network signal extracted from audio recordings against a reference ENF database^3,4^. Such comparisons authenticate characteristics including time, location, and the integrity of recording. Nevertheless, these approaches are confronted with challenges: Dependence on authorized reference ENF databases is limited by legal constraints, the comparison process with large databases is time-consuming, and the effectiveness of these algorithms is reduced for brief audio segments where the uniqueness of the ENF signal fades quickly.

Given the limitations of audio tampering detection relying on ENF reference databases, researchers are exploring alternative methods that are independent of such dependencies. Nicolalde et al. introduced blind forensic techniques for authenticating audio tampering, proposing methods based on phase changes or spectral distance of the ENF signal^5^. They automated authenticity assessment by establishing a categorization threshold, but manual threshold settings were not precise enough. The advent of deep convolutional neural networks provides a compelling alternative, enabling the automatic extraction of latent features from audio without enabling the. Mao et al. put forward a two-dimensional convolutional neural network for binary classification of raw and tampered audio^6^. However, their approach focused solely on the fundamental ENF wave, and neglected the higher-order harmonic components, thereby omitting some distinctive characteristics.

Numerous studies have highlighted the vulnerability of embedded ENF signals in audio recordings to external noise contamination^7^. This issue becomes particularly pronounced in recordings involving human speech , where the lowest fundamental frequency can reach down to 80 Hz, or when music is present, utilizing the same 30 to 500 Hz frequency range that coincides with ENF signals—often exploited for bass and rhythm effects. Such noise interference within the ENF is a critical concern in audio forensics, as it can significantly affect the accuracy of forensic result. To mitigate this challenge, researchers have been developing effective methods for controlling ENF signal noise during audio progressing, which are critical in environment with low Signal-to-Noise Ratio (SNR) scenarios, where many forensic algorithms struggle to provide accurate detection results. Robust noise control techniques specific to ENF signals in audio are essential for the reliability and precision forensic analysis.

The existing research on audio tampering detection mostly focuses on binary classification of original and tampered audio, without considering common tampering techniques such as deletion, insertion, and replacement of audio clips. This study aims to investigate these prevalent tampering techniques by proposing a novel four-class tampering identification problem, which includes untampered audio, deleted audio, inserted audio, and replaced audio. By considering these four categories, the research broadens the scope of audio tampering detection beyond the traditional binary classification. Additionally, it is worth mentioning that the four-class problem can be simplified into a general binary-class tamper detection problem-untampered audio and tampered audio- when the three tampering techniques are consolidated into a single class of tampered audio.

In response to the aforementioned challenges, we have undertaken targeted research efforts. Our main contributions are as follows:

- We designed a four-classification audio tampering detection scenario for the first time, which is able to differentiate between three types of tampering, namely deletion, insertion and replacement, as well as untampered audio, which is richer than the traditional two-classification scenario and more in line with the practical application requirements. This design extends the research scope of audio tampering detection and provides a new perspective for the field of audio forensics.
- We proposed ENR, a noise suppression method for ENF signals that combines the variational modal decomposition (VMD) and the robust filtering algorithm (RFA). the VMD effectively separates and removes the noise components of ENF signals, while the RFA suppresses the power grid noise. This combination method significantly improves the quality of the ENF signal and lays the foundation for subsequent audio tampering detection.
- We proposed a new feature combination method, using the fundamental and its harmonic components of the ENF signal as input features. Through this combination, the model can learn richer feature information, which significantly improves the performance of audio tampering detection. This innovative point verifies the effectiveness of multi-feature combination input and provides a new idea for audio tampering detection.

## Related work

The integration of digital recording systems with power frequency has sparked extensive research on audio tampering identification based on electric network frequency (ENF), which has garnered considerable attention in the industry. Currently, ENF-based approaches for audio tampering identification can be broadly classified into two categories: ENF database-based methods and blind detection-based methods.

### ENF database-based

The foundational link between ENF and the authenticity of digital voice was first established by the Romanian scholar Grigoras^2^, who proposed the ENF criterion for verifying the authenticity of digital voice by extracting the fundamental component of the electric network from the recording and comparing it with a standardized ENF database to determine the authenticity. Liu et al.^8,9^ emphasized the importance of accurate frequency estimation methods and reliable frequency reference databases in the realm of audio forensics. They employed the Short-Time Fourier Transform (STFT) to estimate the ENF signal within audio files and aligned the estimates with an ENF-based frequency database according to the ENF criterion. The results substantiated the precision of STFT method for ENF extraction. Elmesalawy et al.^10^ introduced a novel approach for constructing a robust ENF reference database utilizing Geographic Information Systems (GIS) and comprehensive frequency measurements across wide areas to increase the matching process’s accuracy. Gerazov et al.^11^ advanced a method for recording high-quality ENF reference signals from the power supply using LabVIEW-based virtual instruments. Hua et al.^4^ analyzed the absolute error in ENF signals when compared to the reference database, a crucial step that facilitates the verification of timestamps in audio data. Various digital tampering techniques such as insertion, deletion, and splicing were employed to validate the algorithm. Chowdhury et al.^12^ developed a multi-class support vector machine (SVM) classification model to authenticate the location of recordings through a detailed analysis of ENF sequences extracted from power and audio recordings. The experimental results demonstrated the efficacy of their approach. Karantaidis et al.^13^ proposed the use of the Matthews Correlation Coefficient (MCC), a statistical rate that measures the quality of binary classifications, to detect tampering in multimedia data by calculating the correlation between the reference ENF signal and the estimated ENF signal. These studies have significantly contributed to the advancement of ENF-based audio tampering identification methods, encompassing aspects such as reference database matching, frequency estimation, geographic information integration, signal recording techniques, and classification models.

### Blind detection-based

Nicolalde et al.^5^ presented an audio tampering identification scheme independent of ENF reference signals. This algorithm assesses the authenticity of audio by examining the spectral distance and phase variation of the ENF signal within the audio, thus enhancing flexibility in tampering detection. It employs a threshold derived from the extent of phase shifts at suspected tampering points to facilitate automatic verification of audio integrity. In 2013, Nicolalde et al.^14^ further discussed the presence of higher harmonics in ENF signals, which emerge from nonlinearities inherent in the recording process. When the ENF fundamental signal is corrupted, the phase information around its harmonic frequencies can also be utilized for audio tampering forensics. Esquef et al.^15^ identified audio tampering by comparing the ENF variation around the nominal frequency against the upper limit of normal variation observed in unedited signals, using resulting equal error rate (EER) values as a measure. Reis et al.^16^ proposed an ENF estimator called ESPRIT-Hilbert, which spots outliers in the ENF signal based on abnormal changes observed in the audio recording. The estimation results are then used as inputs to a support vector machine to confirm the presence of tampering. Whilst expediting detection, this technique may risk overlooking certain feature information that could be critical for a comprehensive analysis. Wang et al.^17^ utilized the discrete Fourier transform of the audio signal and a support vector machine classifier to analyze the consistency of ENF components to identify audio tampering. However, the accuracy of this scheme failed to achieve the desired level. Jadhav et al.^18^ proposed a CNN-based audio splicing detection , which excels at extracting high-level features by processing the spectrogram of audio data directly. The study conducted experiments on audio data tampering detection by inserting with 1-s, 2-s, and 3-s segments, whose results showed the highest detection accuracy for 3-s insert tampering. Mao et al.^6^ proposed a two-dimensional convolutional neural network model for binary classification of original audio and tampered audio. Zeng et al.^19^ proposed an audio tampering detection method based on ENF phase sequence representation learning. Zeng et al.^20^ proposed a new method for digital audio tampering detection based on ENF deep spatio-temporal features. However, their approach only considers the ENF fundamental wave as a feature instead of including higher harmonic components as input. Furthermore, the noise reduction methods they designed for denoising progress were not effective enough.

## Problem interpretation

### Four-class tampering detection scenarios

Audio tampering can be executed through deletion, insertion, and replacement, which are classified as deletion tampering, insertion tampering, and replacement tampering, respectively. Much of the existing research focuses on a binary classification problem, that is, distinguishing between original and tampered audio, and it can only determine whether the audio has been tampered with. It would be more helpful for the court to determine the truth if the exact audio tampering technique can be accurately identified. Therefore, this work analyzes these three typical tampering techniques as well as the type of untampered audio and for the first time puts forth a four-class tampering identification problem. Additionally, the four-class problem can be simplified into a universal two-class tamper detection problem by combining the three tampering types into a single category of altered audio. The descriptions of the two classification scenarios are shown in the Table 1 below.

**Table 1:** Description of two classification scenarios.

Classification scene			Type	
Four classes of tampering detection scenarios	Original audio	Deletion tampering	Insertion tampering	Replacement tampering
Two classes of tampering detection scenarios	Original audio		Tampered audio	

### Characteristics of ENFs in tampered audio

Assume a sinusoidal signal with a fixed frequency such as Eq. (1):

1 $$\begin{aligned} x=\partial \cos \left( \frac{2 \pi f }{f_s}+\varphi _{0}\right) , \end{aligned}$$

where ${\partial }$ represents fixed amplitude, *f* represents frequency, $f_{s}$ represents sampling frequency, ${\varphi _{0}}$ represents initial phase.

The correlation between adjacent frames in an audio signal is a crucial characteristic for audio tampering identification. Tampering, such as sound insertion or deletion, can disrupt the natural correlation, causing signal discontinuities or singularities. In the context of ENF-based audio tampering identification, the embedded electric network signal also exhibits similar tampering patterns. The electric network signal, being a pseudo-sinusoidal time-stamped signal that varies over time, will be affected by any tampering operations performed on the audio, such as insertion or deletion. For instance, when a sinusoidal signal is edited, the amplitude before and after the editing positions may experience abrupt changes, resulting in a discontinuous signal. This disruption in the continuity of the electric network signal can indicate potential tampering in the audio. Therefore, identifying audio tampering can be reframed as detecting continuity or discontinuity in the embedded electric network signals. The regions of tampered audio can thus be identified by analyzing the correlation and continuity of the electric network signal. Note that the specific equation referenced as “formula (1)” is not available in the provided text.

The description provided explains three common tampering methods: deletion tampering, insertion tampering, and replacement tampering. The latter two methods involve altering the original audio and result in specific characteristics in the ENF signal, while Deletion tampering involves removing a segment of the original audio at a random location.

**Figure 1:**
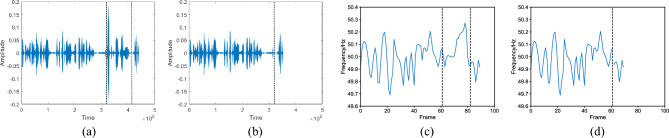
An example of deletion tampering. (**a**) Original audio; (**b**) tampered audio; (**c**) ENF signal of original audio; (**d**) ENF signal of tampered audio.

The comparison diagrams in Fig. 1a,b show the audio before and after deletion tampering, while the corresponding ENF diagrams in Fig. 1c,d illustrate the ENF signal before and after tampering. After deletion tampering, a point of discontinuity appears in the ENF signal. Insertion tampering, on the other hand, entails inserting audio clips with the same sampling rate as the original audio. at a random position, resulting in a different kind of tampering effect.

**Figure 2:**
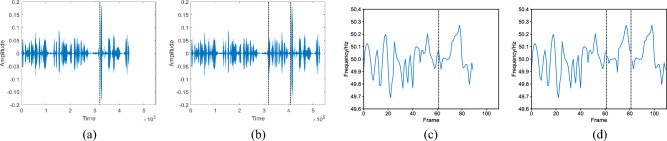
An example of insertion tampering. (**a**) Original audio; (**b**) tampered audio; (**c**) ENF signal of original audio; (**d**) ENF signal of tampered audio.

The comparison diagrams in Fig. 2a,b display the audio before and after insertion tampering, while the corresponding ENF diagrams in Fig. 2c,d depict the ENF signal before and after tampering. In the case of insertion tampering, two points of discontinuity can be observed in the ENF signal. As for replacement tampering, it involves exchanging a specific segment at a random position of the audio in sequential.

**Figure 3:**
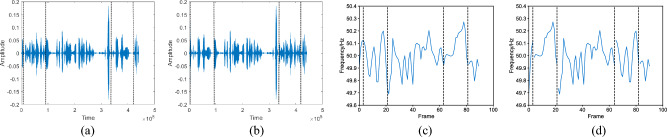
An example of exchange tampering. (**a**) Original audio; (**b**) Tampered audio; (**c**) ENF signal of original audio; (**d**) ENF signal of tampered audio.

The comparison diagrams in Fig. 3a,b showcase the audio before and after replacement tampering, while the corresponding ENF diagrams in Fig. 3c,d demonstrate the ENF signal before and after tampering. In the ENF signal after replacement tampering, four points of discontinuity can be identified. By analyzing the discontinuities in the ENF signal caused by different tampering methods, it becomes possible to detect and identify the specific tampering technique employed to tamper with the audio.

## Algorithm design

**Figure 4:**
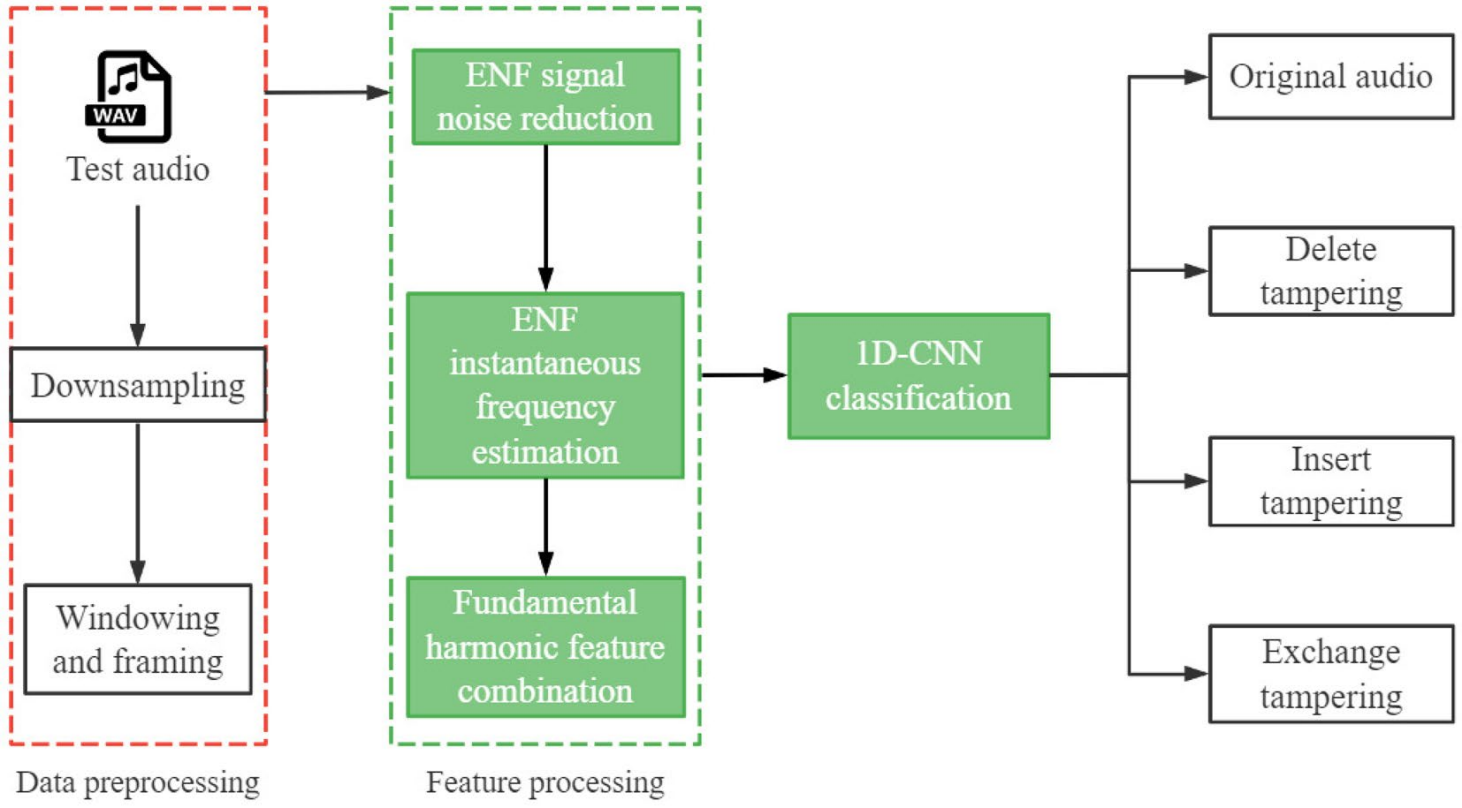
ATD framework structure.

The ATD framework we propose is mainly composed of data preprocessing, feature processing, network model building, and final tampering identification and classification. Data downsampling and data windowing are the two primary steps in data preprocessing. Feature processing mainly includes noise reduction of ENF signal in the audio to be verified, ENF signal estimation, and combination of fundamental harmonic features. The network model construction is mainly based on the 1D-CNN network model to process the one-dimensional ENF signal data. The final tampering detection classification aims to effectively identify the four-classs detection scene (or binary-class detection scene when simplified) scene proposed in this study. The overall framework of ATD is illustrated in Fig. 4) below.

### Data preprocessing

In processing audio samples, the initial step involves downsampling and windowing. This ensures a consistent number of sampling points for the ENF signal within each cycle, and reduces the computational load of subsequent data analyses. The downsampling of the audio data is performed to achieve these goals.

In audio signal processing, short-time analysis technique is widely used for feature extraction of speech and audio signals. This technique is used to analyse the changing characteristics of a signal over a short period of time by performing a frame-splitting process on the signal. Thus, to prepare for such analysis, it is necessary to segment the signal into shorter frames it. This entails dividing the longer speech signal into multiple segments, often with overlapping portions to ensure smooth transitions between consecutive frames. If we denote the total length of a voice signal as *Len*, the formula for calculating the frames can be expressed as shown in Eq. (2):

2 $$\begin{aligned} f_n=(Len-wlen+inc)/inc, \end{aligned}$$

where $f_n$ represents the number of frames after framing, *wlen* represents the frame length of each frame, *inc* represents the frame shift, $n=1,2,...,wlen$, the overlapping part $overlap=wlen-inc$

**Figure 5:**
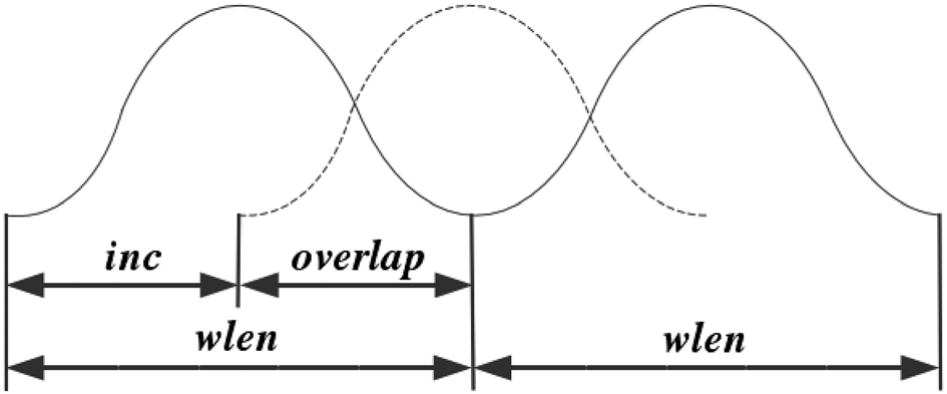
Framing diagram of speech signal.

The speech signal framing process is shown in Fig. 5.

### Feature processing

#### ENF signal noise reduction

The ENF signal that is embedded in the audio recording is weak relative to the noise, making the assessment of the ENF signal highly susceptible to noise interference. To address this issue, we propose a noise reduction method for the ENF signal, termed ENR, which utilizes the VMD algorithm^21^ and RFA algorithm^7^. The accuracy of any ENF instantaneous frequency estimation technique can be indirectly improved by passing the noisy ENF signal through the noise reduction algorithm. Let*x*(*n*) represents the audio signal to be verified, and the general signal model is formulated as shown Eq. (3):

3 $$\begin{aligned} \tilde{x}(n)=\tilde{s}(n)+\tilde{c}(n)+\tilde{v}(n), \end{aligned}$$

where ${\tilde{s}(n)}$ represents ENF signal, ${n \in \{0,1, \ldots \ldots , N-1\}}$, ${\tilde{c}(n)}$ represents the audio noise interference signal, ${\tilde{v}(n)}$ represents the electric network disturbance signal.

**Figure 6:**
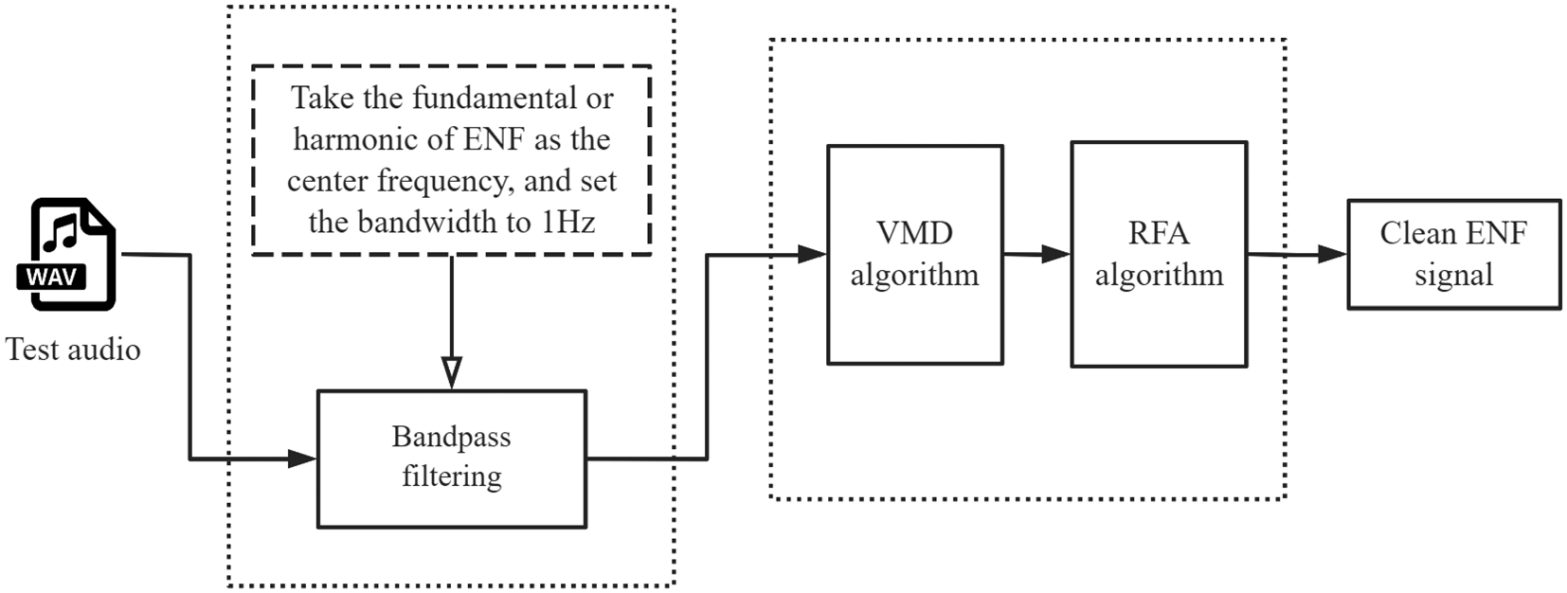
ENF signal noise reduction process.

In the noise reduction process depicted in Fig. 6, the ENR method initially employs a band-pass filter to filter out unwanted out-of-band audio content and electrical network interference signals, and obtains an in-band signal model *x*(*n*), which is formulated as ${{x}(n)={s}(n)+{c}(n)+{v}(n)}$. In order to extract the pure ENF signal, namely *s*(*n*), it is necessary to eliminate the audio noise interference signal *c*(*n*) and the electric network interference signal *v*(*n*). ENR comprises two primary algorithms: VMD algorithm and RFA algorithm. After the application of the VMD algorithm, the audio noise interference signal *c*(*n*) in the ENF signal can be effectively filtered out. Consequently, the signal model after filtering out the audio noise interference signal is represented as ${x(n)=s(n)+v(n)}$. However, in actual signal processing, the separation between noise and non-stationary, nonlinear, and signals cannot be fully realized solely through the use of the VMD algorithm^22,23^. Therefore, To achieve a better noise reduction result, the signal must undergo additional processing using supplementary noise reduction techniques. The RFA algorithm^7^, which functions as a electric network noise reduction mechanism, can effectively suppress the additional electric network interference noise *v*(*n*). Integrating the RFA algorithm with the VMD algorithm proves advantageous in isolating a purer ENF signal *s*(*n*).

It is important to acknowledge that numerous audio recording devices and software available in the market have the capability to modify the output frequency of multimedia data. For instance, the Tascam DR-07X offers the option to selectively attenuate frequencies below 40, 80, or 120 . Similarly, the Sony ICD-TX650 digital recorder has a frequency response range spanning from 95 to 20,000 Hz. Additionally, many smartphone recording applications provide options to select a frequency range or automatically apply low-frequency filters. Consequently, the application of ENF fundamental frequency-based research methods for audio authenticity analysis is not always feasible. In related literature^24^, Hajj-Ahmad proposed the utilization of higher harmonic components for ENF estimation, although the research was limited to the examination of a single harmonic component. A thorough analysis of the current methodologies indicates that the Enhanced Noise Reduction (ENR) technique is capable of effectively removing noise from both the fundamental and higher harmonic bands of the ENF signal

#### Feature combination

In this study, we adopt a direct modeling approach where the ENF signal, extracted via an instantaneous ENF estimation algorithm, serves as the network input feature. This approach helps to mitigate information loss associated with the extraction of representative feature values. By directly using the ENF signal extracted from the audio data as the input feature, we aim to simplify the process of audio forensics identification. Previous research has indicated that the ENF signal manifests not only at its fundamental frequency but also within its higher harmonics. The strength of these higher harmonics vary with the recording environment and technology employed, yet their fluctuation characteristics align with the fundamental frequency of the ENF^24^. Presently, many ENF-based audio tampering forensics techniques can be circumvented due to the existence of methods^25,26^ that can conceal tampering traces can be concealed by eliminating the fundamental frequency of the ENF signal from the audio. Consequently, the fundamental component may often be absent in practical scenarios. In such cases, when identifying audio tampering, it is crucial to consider the higher harmonics of the ENF, as these elements may still be present and indicative of manipulation.

**Figure 7:**
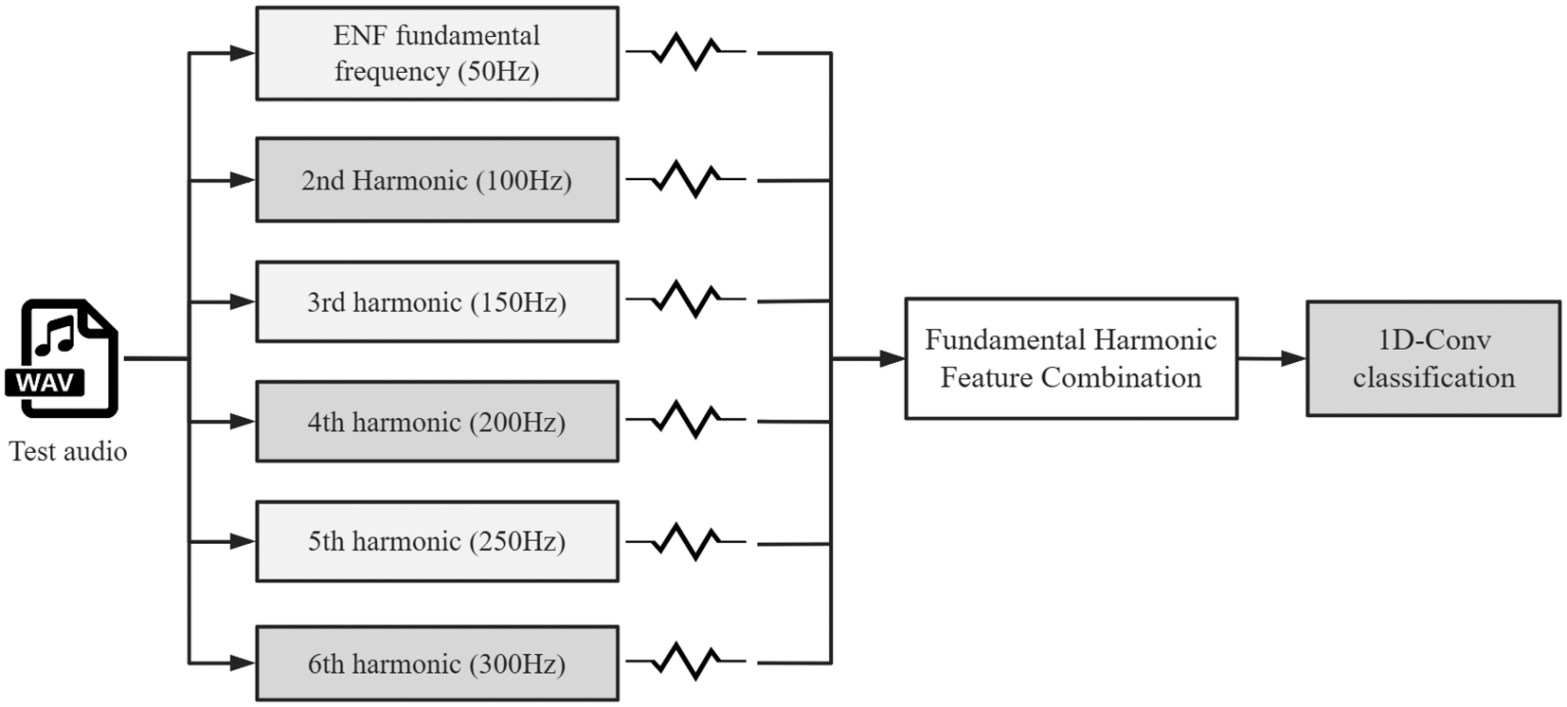
ENF fundamental harmonic feature combination.

In light of the aforementioned factors, this research seeks to develop a technique that incorporates fundamental harmonic features to enhance the model’s signal feature learning efficiency. This technique, referred to as the Feature Combination Method (FCM), aims for precise signal characteristic capture via integration of the fundamental waveform and its higher harmonic orders, which is illustrated in Fig. 7. This method aims to achieve a more robust and precise feature representation by combining different components present in the ENF signal, rather than relying on a single feature component. The FCM leverages spectrograms centered on multiple harmonics and the ENF fundamental to construct multiple input channels for the network. Firstly the FCM method enables the model to learn richer signal features by using the fundamental of the ENF and its harmonics as multiple input channels. Compared to using only the fundamental of the ENF, combining multiple harmonic components enables the model to learn more diverse signal features, thus improving the feature learning efficiency. The harmonic components contain signal information that is complementary to the fundamental, which helps to improve the feature learning ability of the model.

### Network model

**Figure 8:**
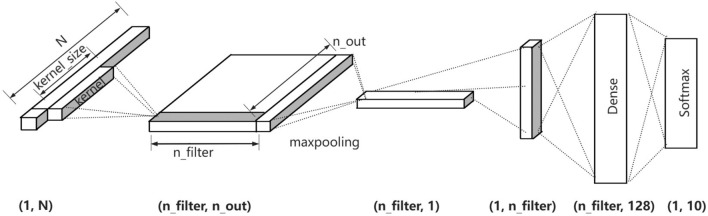
1D-CNN network model.

Since the ENF signal in the input network is a one-dimensional time series data, it can be read using a one-dimensional convolutional neural network. The translation invariance properties of the CNN network can also be fully exploited to learn the tampering features of the signal data due to the ambiguity of the tampering position. Therefore, this study designs a one-dimensional convolutional neural network model (1D-CNN) that employs convolution akin to scanning the signal from left to right with a small window. In the model, the feature data needs to be compressed by the pooling layer, which can minimize the quantity of features, and reduce the phenomenon of overfitting. The model also employs max pooling, a technique that selects the maximum value within a defined region to represent the feature value of that area. The final compressed feature set is then fed into a fully connected layer for classification purposes. The specific network model structure is shown in Fig. 8. It can be seen from the network model diagram that the size of the convolution kernel must not exceed the length of the input ENF signal *N*, and the input data undergoes convolution with the designated number of convolution kernels. During the convolution process, the dimensions of the training set must align to maintain dimensional consistency. To achieve this, the commonly used Padding method is employed to supplement the data. When the input signal length is less than *N*, padding is performed using the electric network center frequency of the corresponding fundamental wave or harmonic.

The key experimental configurations in this study consist of binary-class and four-class tampering detection. The binary-class approach ascertains the presence of alterations in the audio, whereas the four-class method identifies both the presence and the type of alterations. Building on the preceding analysis, the dimension of the output result can be determined as shown in Eq. (4):

4 $$\begin{aligned} {{n}_{-}{out}}=\frac{{N}-{ {kernel}_{-}{size}}}{S}+1, \end{aligned}$$

where ${{n}_{-}{out}}$ represents the dimension of the data output after convolution, *N* represents the length of the input data, ${kernel}_{-}{size}$ represents the convolutional kernel, *S* represents the step size of the convolution kernel’s movement. The specific details of the entire convolution process and its associated dimensions are shown in Fig. 8. The 1D-CNN model leverages its translation invariance property to perform a one-dimensional convolutional scan on the dataset, subsequently employing a max-pooling layer to distill the post-convolution data. Through extensive training on a substantial dataset, the model is capable of learning the optimal features for detecting tampering.

## Experiments setup

The Carioca 1 dataset^27^, an open-source collection recorded via landlines, comprises 200 audio clips sampled at 144.1 kHz with a 16-bit quantization depth for single-channel waveforms. The dataset includes an equal number of original (50 male and 50 female voices) and tampered audio files, each ranging from 30 to 40 seconds in duration. The Electric Network Frequency (ENF) of the fundamental wave is maintained at 50 Hz. Audio files with names terminating in “e.wav” indicate tamperings. Accompanying documentation provides detailed descriptions of the tampering methods and the specific alterations made to each audio file.

To mitigate the limitations imposed by the limited sample size in the Carioca 1 dataset, we employed a data augmentation strategy. This strategy involved segmenting the 100 original audio tracks from the corpus into 10-s clips. These clips were extracted at random intervals, allowing for each original audio to yield 10 distinct segments. As a result, we generated a total of 1000 new, unaltered audio samples, each with a duration of 10 s. This expanded dataset comprises an equal distribution of 500 male and 500 female voice samples.

Before the tampering detection experiment, the supplementary 1000 original audio clips requires manual modification to ensure a balanced number of samples across each classification category. In the four-class tamper detection model, we manually modify the 1000 samples four times, each time using a different tampering technique to ensure that there are 1000 samples in each category to achieve a 1:1:1:1 sample ratio. Specifically, we performed four separate processes of deletion, insertion, replacement, and no tampering on each original sample so that there were 1000 samples in each category. In the binary-class tamper detection model, we similarly ensure that the number of original audio samples and tampered audio samples are balanced. Therefore, we retained 1000 original audio samples and tampered the other 1000 original samples with three kinds of tampering: deletion, insertion, and substitution, so that the number of tampered samples reached 1000, achieving a 1:1 sample ratio. With such manual modifications, we ensured that each category had a sufficient number of samples, thus avoiding the impact of sample imbalance on model training and improving the generalisation ability of the model. Tables 2 and 3 present a comprehensive breakdown of the sample categories and their respective counts:

**Table 2:** Four classes of tampering identification.

	Number of samples/piece	Label
Original audio	1000	“0”
Deletion tampering	1000	“1”
Insertion tampering	1000	“2”
Replacement tampering	1000	“3”

**Table 3:** Two classes of tampering identification.

	Number of samples/piece	Label
Original audio	1000	“0”
Deletion tampering	333	“1”
Insertion tampering	333	“1”
Replacement tampering	334	“1”

### Evaluation indicators

In order to assess the generalization ability of the model, appropriate evaluation metrics must be applied. For the four-class tampering detection scenario, the accuracy serves as the primary metric. For binary tampering detection scenarios, precision, recall, and the F-Score—the harmonic mean of precision and recall—are utilized for evaluation. In the binary-class framework, the label of the original audio is set to “0”, and the label of the tampered audio is set to “1”. TP (True Positive) indicates the number of tampered audio samples that correctly identified by the model as tampered. FP (False Positive) indicates the number of original audio samples wrongly classified as tampered by the model. FN (False Negative) represents the number of tampered audio sample incorrectly detected as original. TN (True Negative) represents the number of original audio samples accurately recognized as original. Using these four variables, the following four evaluation indicators can be calculated.

- *Accuracy* indicate the ratio of correctly classified samples, both original and tampered, to the total number of samples examined.
  5 $$\begin{aligned} \text { Accuracy }=\frac{T P+T N}{T P+F P+T N+F N}. \end{aligned}$$
- *Precision* indicates the probability that a sample predicted as tampered is indeed tampered, essentially measuring the correctness of positive predictions.
  6 $$\begin{aligned} \text { Precision }=\frac{T P}{T P+F P}. \end{aligned}$$
- *Recall* indicates the proportion of actual tampered samples that are correctly identified as such.
  7 $$\begin{aligned} \text { Recall }=\frac{T P}{T P+F N}. \end{aligned}$$
- *F-Score* represents the harmonic mean of precision and recall.
  8 $$\begin{aligned} \text { F-Score }=2 \cdot \frac{ \text{ Precision } \cdot \text{ Recall } }{ \text{ Precision } + \text{ Recall } }. \end{aligned}$$

For the evaluation metrics of noise reduction algorithms for electric frequency signals, this chapter quantifies their noise reduction performance through Normalized Misalignment (NM) and correlation coefficient (CC), the corresponding equations are as follows:

9 $$\begin{aligned} \text{ NM }= & {} \frac{\sum _{n}(f^{'}(n) - f_{GT}(n))^2}{\sum _{n} f_{GT}^2(n)}, \end{aligned}$$

10 $$\begin{aligned} \text{ CC }= & {} \frac{\sum _{n}[(f^{'}(n) - \hat{\mu })(f_{GT}(n) - \mu _{GT})]}{\sqrt{\sum _{n}(f^{'}(n) - \hat{\mu })^2}\sqrt{\sum _{n}(f_{GT}(n) - \mu _{GT})^2}}, \end{aligned}$$

where $f_{GT}(n)$ denotes the reference ENF signal (Ground Truth), $f^{'}(n)$ denotes the estimated signal after noise reduction by the signal noise reduction algorithm in this chapter, $\mu _{GT}$ and $\hat{\mu }$ denote the corresponding sample means. Note that NM $\ge$ 0, where smaller results represent better performance, and CC $\in [-1, 1]$, where larger results represent more correlation between the two signals.

### Experimental details

#### Experimental environment

In the experiment, the operating system is Windows 10, the compiler is MATLAB 2018A and PyCharm, the experiments were conducted using Python 3.7 and PyTorch 1.8.1 with CPU support, the machine learning library sklearn 0.0, and the data analysis library is Matplotlib 3.0.3.

#### Experimental setup

In the data preprocessing phase, the new sampling frequency of down-sampling is set to 20 times the fundamental frequency of ENF. The audio data that to be analyzed, for instance, is down-sampled to 1 kHz if the ENF pivot value is 50 Hz. The Hamming window is selected as the window function. The frame length *wlen* is set to 30 ms, and the frame shift *inc* is set to 15 ms. The overlap between frames is set to 50% to ensure smooth transitions.

In the preprocessing of the audio data, band-pass filtering is tailored to the center frequency of the targeted fundamental or harmonic. Next, the ENR method is utilized to eliminate noise and electrical network interference signals. The STFT algorithm is employed by the instantaneous ENF estimate technique. The data length *N* in the two different experimental scenarios in this study is uniformly set to 350. This implies that the audio data is segmented into *N* frames, with each frame yielding an individual instantaneous ENF frequency estimate. To ensure uniformity in input data length, signal padding is applied to signals shorter than *N*. In this study, the bit complement is chosen to be the precise center frequency value matching to the electric network signal. For example, for the existing second harmonic signal data whose length is *L*, $(N-L)$ instances of 100 Hz need to be padded to achieve a consistent input signal length of *N*. Then, the feature matrix is constructed using the fundamental harmonic feature combination method, which is then fed into the 1D-CNN network model. The training and testing datasets in this study are evenly split into a ratio of 5:5 to enhance the accuracy of the results. After processing through the 1D-CNN model, the Softmax layer yields a probability distribution for each category, which helps to determine the type of audio tampering.

The hyperparameter configuration in the neural network can profoundly influence the detection outcomes. For example, critical parameters such as learning rate, convolution kernel size, number of filters, etc. In order to determine the best hyperparameter settings for the 1D-CNN network, this section evaluates the impact of these parameters using the basic wave feature component as an example. In both supervised learning and deep learning, the learning rate plays a crucial role in determining whether and how swiftly the objective function converges to its minimum value. Tables 4 and 5 respectively present the results of the detection indicators for four-class tampering detection and binary-class tampering detection under different learning rates.

The four-class tampering detection mainly uses the accuracy rate as the evaluation index. It can be seen from the Table 4 that the 1D-CNN model attains its peak accuracy at a learning rate of 0.05. In contrast, binary-class tampering detection, as detailed in Table 5, shows superior accuracy, precision, and F-Score relative to other parameter configurations when the learning rate is set to 0.1. Although the corresponding recall rate of the model is not the highest, the F-Score can better reflect the real situation of the model. According to the experimental results of the model under different learning rates, the learning rate in the four-class scenario is set to 0.05, and the learning rate in the two-class scenario is set to 0.1.

**Table 4:** Single-feature component four-class tampering detection model results with different learning rates (feature selection 50 Hz).

Measure	Accuracy (%)
0.1	82.9
0.05	85
0.01	84.75
0.005	81.25
0.001	81.75
0.0005	77.65
0.0001	79.6

**Table 5:** Single-feature component binary classification tampering detection model results with different learning rates (feature selection 50 Hz).

Measure	Accuracy (%)	Precision (%)	Recall (%)	F-Score (%)
0.1	82.5	87.47	77.15	81.99
0.05	82.4	83.79	78.87	81.22
0.01	81.9	81.48	79.84	80.65
0.005	81.5	81.34	81.01	81.17
0.001	79.1	77.65	79.84	78.73
0.0005	76.6	76.37	75.91	76.14
0.0001	74.6	72.71	76.59	74.6

**Figure 9:**
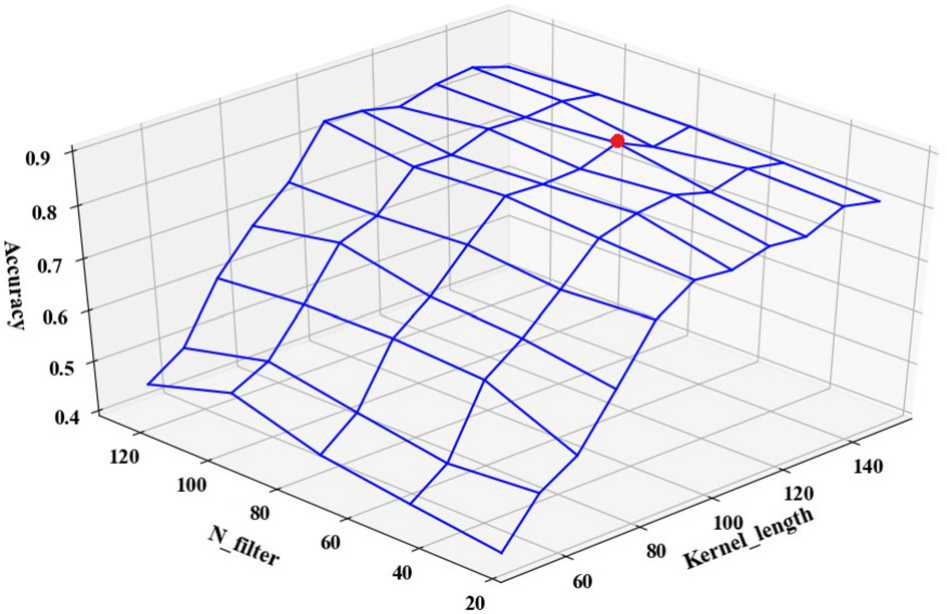
The influence of the size of the convolution kernel and the number of filters of a single feature component on the accuracy of the model in the four-classification scenario.

**Figure 10:**
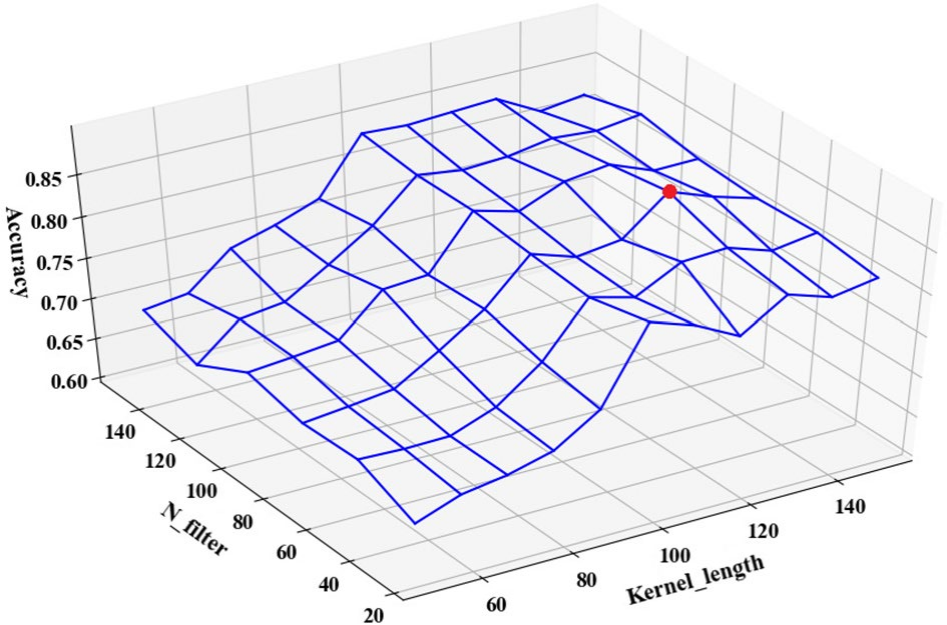
The influence of the size of the convolution kernel and the number of filters of a single feature component on the accuracy of the model in the two-classification scenario.

Figures 9 and 10 respectively illustrate the classification accuracy of four-class and binary tampering detection under different number of filters and different size of convolution kernel. The Figs. 9 and 10 indicate that both the kernel size and the number of filters significantly impact the model’s performance, with the locations marked by red dots representing the configurations that achieved the highest accuracy. It is observed that the smaller the convolution kernel size and the filter number, the poorer the accuracy, as such settings may limit the model’s capacity to learn and distinguish effectively between tampered and untampered features. Additionally, Fig. 9 reveals that an excess of filters can also deteriorate the model’s accuracy, as it will lead to complex model and overfitting issues. As a result, it’s important to select the appropriate convolution kernel size and number of filters. This work determines its size based on empirical experience and analytical evaluations. Optimal performance is achieved for both binary and four-class tamper detection when the number of filters is set to 75 and the convolution kernel size is set to 130, according to testing results. Therefore, in these two classification scenarios, the 1D-CNN model is configured with 75 filters and a convolution kernel size of 130.

The configuration of the 1D-CNN model are summarized in Table 6. The model employs stochastic gradient descent (SGD) for optimization and incorporates the rectified linear unit (ReLU) as its activation function, a prevalent choice in neural network design. This work uses the cross-entropy function as its loss function, which will continuously optimize the network parameters and weights to refine the model. In the experimental section, the 1D-CNN model is contrasted with many other machine learning models, such as the Support Vector Machine (SVM), Neural Networks (NN), and Random Forest (RF), etc. The results of these comparative experiments show that the 1D-CNN model has more advantages in processing time series data. The parameter settings for the relevant models are also summarized in the Table 6.

**Table 6:** Model training parameter setting table.

Model	Parameter settings
1D-CNN	Activation function: Relu
	Optimization: SGD
	Loss function: Cross entropy
	Epoch: 2000
	Batch size: 32
	Stride: 1
	Pooling: Max pooling
	Hidden layers: 128 dimensions
Random Forest	Trees: 30
	Depth: 8
	Sampling: With replacement
SVM	Kernel: RBF
Neural network	Activation: Relu
	Iterations: 5000
	Hidden layers: 128 dimensions

## Results and discussion

### Results of the ENF noise reduction method

**Figure 11:**
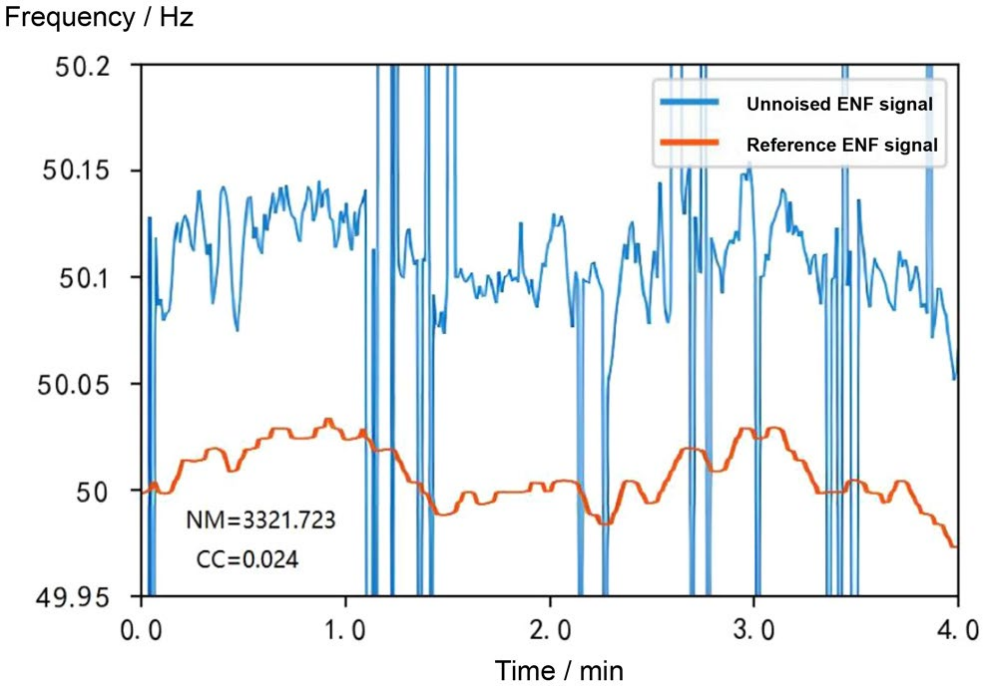
Comparison of the uncancelled ENF signal with the reference ENF signal.

**Figure 12:**
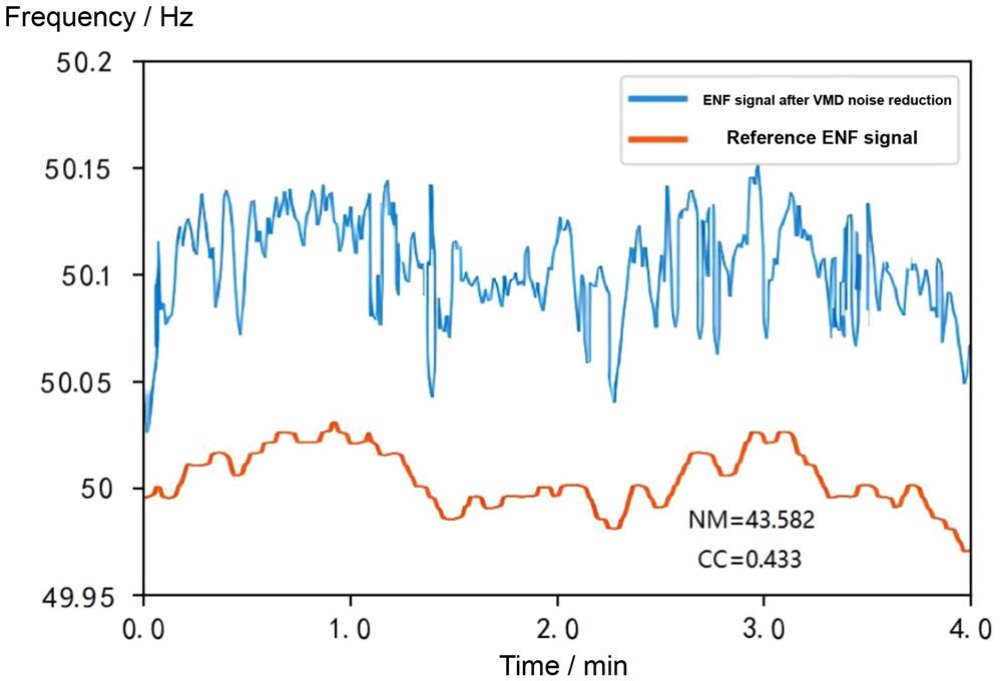
Comparison of ENF signal after noise reduction by VMD algorithm with reference ENF signal.

**Figure 13:**
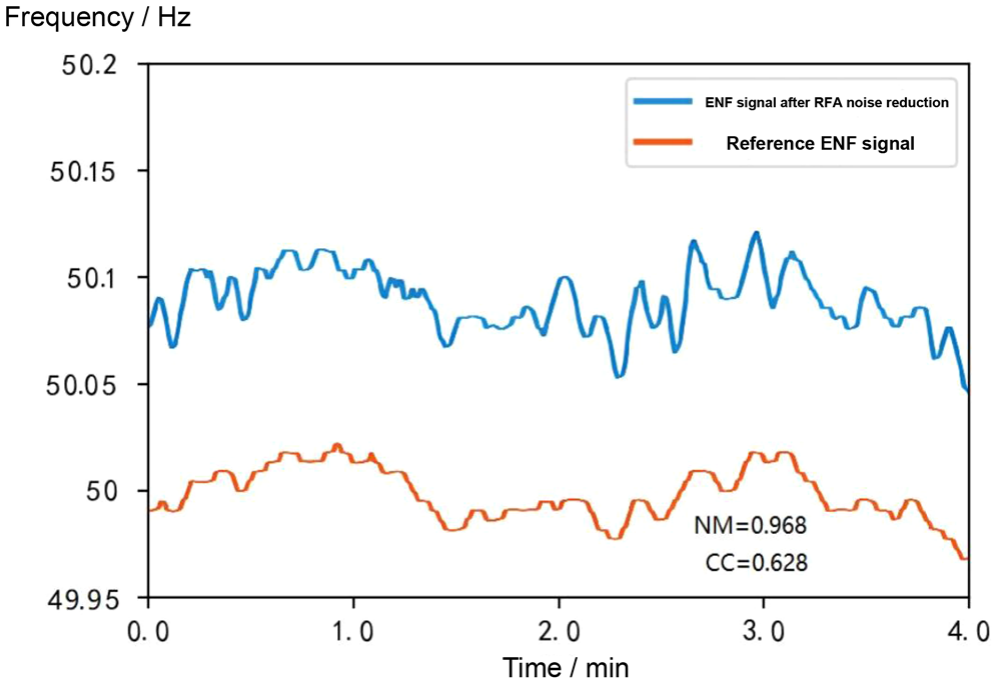
Comparison of ENF signal after noise reduction by RFA algorithm with reference ENF signal.

**Figure 14:**
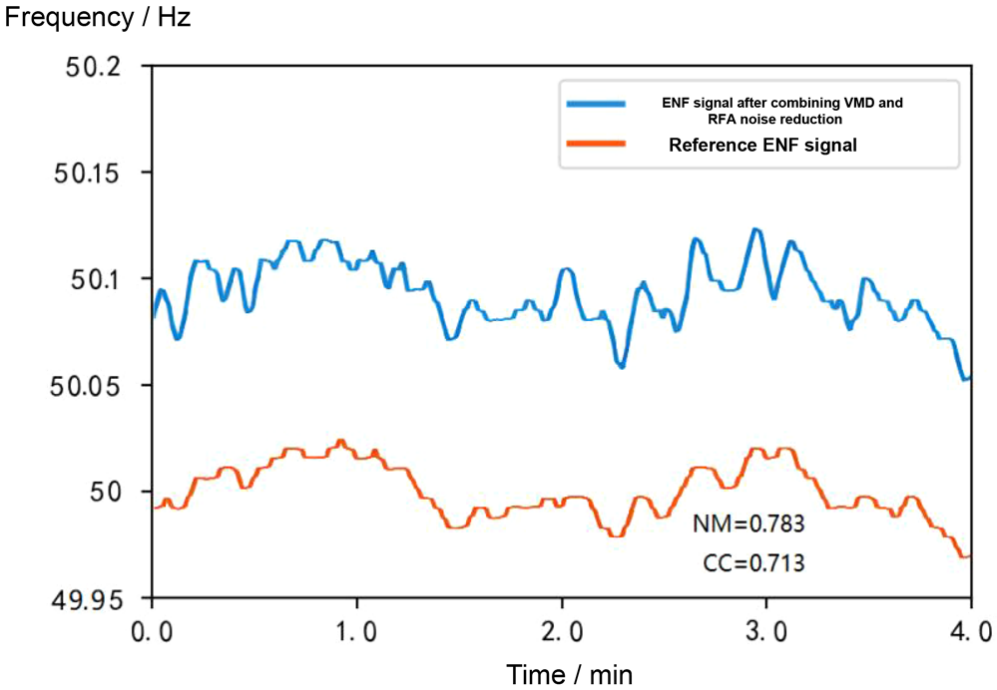
Comparison of the ENF signal after combining the VMD and RFA algorithms for noise reduction with the reference ENF signal.

In this subsection, synthetic signal simulation experiments are designed to verify the noise reduction performance of the algorithms in this chapter, with an audio length of 4 minutes. The detailed results of the synthetic signal experiments are shown in Figs. 11, 12, 13 and 14. The ENF signal extracted directly without noise reduction has an NM of 3321.723 and a CC of 0.024 compared with the reference ENF signal.

As can be seen in Fig. 12, the signal has been subjected to the VMD algorithm, which basically removes the originally observed pseudo-spikes from the signal. Due to the low noise interference in the synthesised signal, the overall characteristics of the ENF remain almost the same except for the removal of the pseudo-spikes in the signal. Compared with the reference ENF signal, the NM of the extracted ENF signal after the VMD algorithm is much reduced and the CC of the extracted ENF signal is also improved, which means the VMD algorithm can reduce the normalised standard deviation of the extracted ENF signal by a very large amount.

The noise reduction results of the RFA algorithm are shown in Fig. 13. Compared with the reference ENF signal, the NM of the extracted ENF signal after the RFA algorithm is 0.968 and the CC is 0.628 and 0.024 respectively, which shows that the RFA algorithm has an outstanding performance in noise reduction for the special signal of ENF.

The VMD algorithm firstly removes the noise interference from the ENF signal, and then the RFA algorithm is applied to the signal to remove the grid interference, in order to further reduce the noise of the grid signal. The results are shown in Fig. 14, where the NM is reduced to 0.783 and the CC is improved to 0.713. The results show that the combination of the RFA algorithm and the VMD algorithm achieves better noise reduction.

### The results and discussion of four-class tampering detection experiment

#### Single feature component experiment

**Figure 15:**
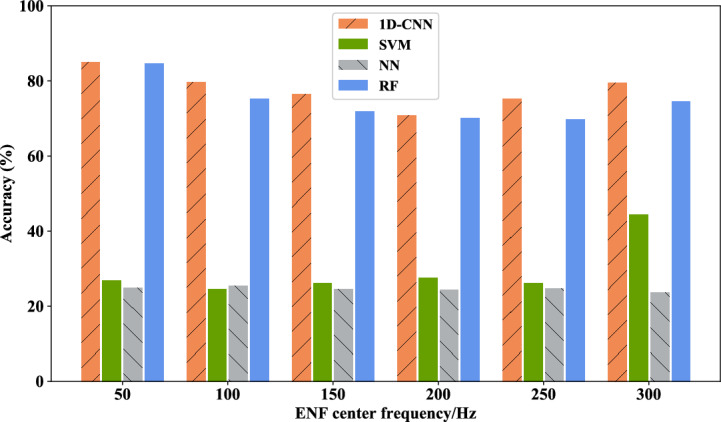
Four-class tampering detection results under single feature component of different models.

**Figure 16:**
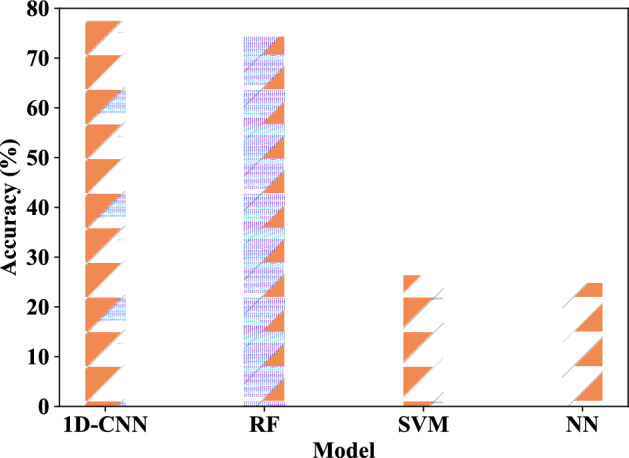
Average accuracy rates of four-class tamper detection under single feature component of different models.

First, the four-class tampering detection scenario proposed in this chapter is tested. This phase involves an examination where solely the fundamental or a specific harmonic component of the ENF signal is employed as the feature input for the model. The experimental component compares the 1D-CNN model with other machine learning models, such as SVM, NN, RF, etc., in order to demonstrate the high performance and efficacy of 1D-CNN. Figure 15 presents the results of the four-class tampering detection accuracy results under the single feature component, Fig. 16 illustrates the average accuracy results derived from different feature components of the four models under the four-class scene. The average accuracy of 1D-CNN is 3.142% higher than that of RF. It can be seen from the experimental results that both models have demonstrated commendable results in this test, where the 1D-CNN model exhibits the best performance, followed by RF. This is because 1D-CNN has better performance on time series data, whereas RF on multi-dimensional data. Conversely, SVM and NN exhibit comparatively weaker outcomes. Table 7 provides a detailed view of the single-component feature audio tampering detection results based on the 1D-CNN model in the four-class scene.

**Table 7:** Single-component feature audio tampering detection results based on 1D-CNN model in four-classification scenarios.

Feature selection (Hz)	Accuray (%)
50	84.95
100	79.75
150	76.45
200	70.75
250	75.3
300	79.55
Average accuracy	77.79

**Figure 17:**
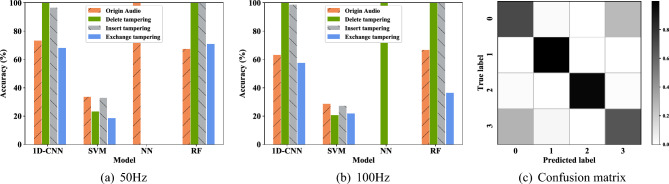
The classification accuracy results of the single feature components of different models in the four-classification scenario. (**a**) 50 Hz; (**b**) 100 Hz; (**c**) Confusion matrix.

Figure 17a,b respectively display the classification prediction accuracies of different models in the four-class scenario when the input features are 50 Hz and 100 Hz. The outcomes reveal that while the 1D-CNN does not achieve the highest prediction accuracy across all four categories, its performance is notably more stable, underscoring its robustness in handling time-domain sequences. In contrast, the predictions of the NN model tend to fluctuate significantly. A confusion matrix diagram of the four-class tampering detection is depicted in Fig. 17c to better illustrate the detection situation of the model for each class.

#### Multi-feature component experiment

In this experiment, the detection accuracy of ENF is improved by combining the fundamental harmonic features. Table 8 shows the four-class detection accuracies of audio tampering based on the 1D-CNN model, along with the specific feature combination methods and experimental results are shown in Table 8.

**Table 8:** Multi-component feature audio tampering detection results based on 1D-CNN model in four-classification scenarios.

Feature selection (Hz)	Accuray (%)
50 + 100	91.95
50 + 100 + 150	93.75
50 + 100 + 150 + 200	89.45
50 + 100 + 150 + 200 + 250	89.25
50 + 100 + 150 + 200 + 250 + 300	86.3

**Table 9:** Single-component feature audio tampering detection results based on 1D-CNN model in binary classification scenarios.

Feature selection	Accuray (%)	Precision (%)	Recall (%)	F-Score (%)
50	88.8	90.35	86.79	88.53
100	90.3	88.39	92.01	90.16
150	88.5	89.82	87.55	88.67
200	84.8	86	83.99	84.98
250	86.4	85.52	86.9	86.2
300	85	85.83	83.79	84.8

The experimental results demonstrate that there is no proportional enhancement in the model’s performance with an increase in the number of feature components. This may attribute to certain damaged high-order harmonic components that cannot be denoised, which will modify the general properties of the signal when combined. The outcomes of the experiments demonstrate that the most effective combination comprises the fundamental wave along with the second and third harmonics. In the four-class scenario, from the single-feature component experiment to the one involving multi-feature components, the classification accuracy of the 1D-CNN model increased from 77.79 to 93.75%. The experimental results of single and multi-feature components demonstrate that, while the classification accuracy is high when each electric network frequency (ENF) harmonic component is processed as an individual input channel, the accuracy further increases when the ENF fundamental and harmonic components are integrated. This combination enables the network model to learn more nuanced features, thereby elevating the classification precision. The results of the experiment support the beneficial impact of the combination of multi-feature components.

### The results and discussion of binary-class tampering detection experiment

#### Single feature component experiment

**Figure 18:**
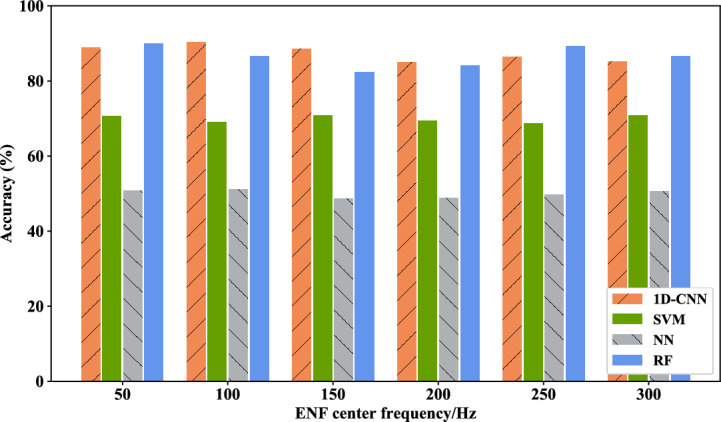
Binary classification tampering detection results under single feature component of different models.

**Figure 19:**
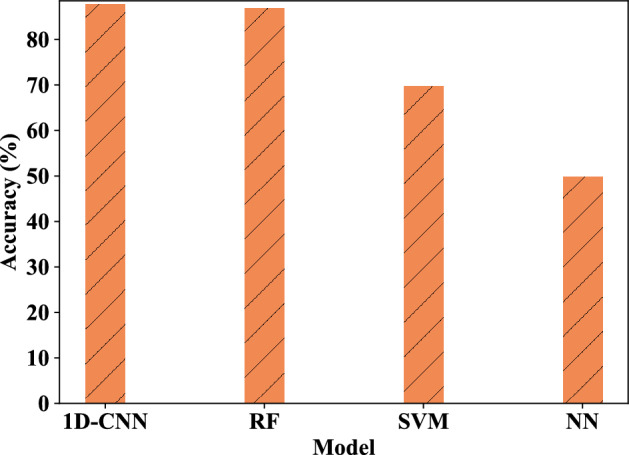
The average accuracy of binary tampering detection under different models with single feature component.

This part of the experiment focuses on the general detection and binary classification of the tampered audio and original audio. In this experiment, only the fundamental signal of the ENF signal or a certain harmonic component serves as a feature input for other machine learning models such as SVM, neural network, and random forest. Figure 18 illustrates the two-class tampering detection accuracy result of each model under the single feature component, while Fig. 19 presents the average accuracy of binary classification utilizing different feature components across the four models, where, the average accuracy of 1D-CNN is 1% higher than that of RF. From the experimental results, it can be seen that the results of 1D-CNN maintains superiority in the binary classification scene. Table 9 details the precision rate, recall rate, F1 value and other results of the 1D-CNN model under different feature components.

**Figure 20:**
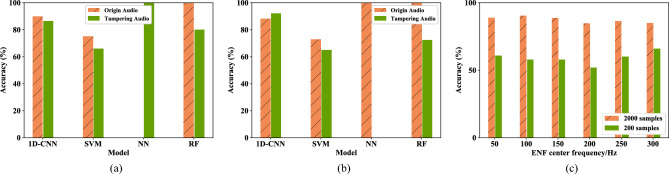
The results of each classification accuracy of the single feature components of different models in the two-class scenario. (**a**) 50 Hz; (**b**) 100 Hz; (**c**) Detection results of single feature components with different sample numbers in binary tampering scenarios.

The Fig. 20a,b respectively represent the classification prediction accuracies of different models in the binary-class scenario when the input feature is 50 Hz and 100 Hz. It can be seen from the results that although the accuracies of 1D-CNN for original audio and tampered audio are not always the highest, it is more stable, and the experimental results verify the 1D-CNN model’s proficiency in handling time-domain sequences.

This study also explores the impact of the sample size on the accuracy of the model. As shown in Fig. 20c, the 1D-CNN model is trained using 2000 samples and 200 samples, respectively. The experimental results show a direct correlation between increased data volume and enhanced model accuracy, revealing the advantages of deep learning.

#### Multi-feature component experiment

In this experiment, the feature input adopts the multi-component method of fundamental harmonic feature combination to improve the extraction accuracy of ENF. The details of the feature combination methods and the corresponding results are shown in Table 10.

**Table 10:** Multi-component feature audio tampering detection results based on 1D-CNN model in binary classification scene.

Feature selection	Accuray (%)	Precision (%)	Recall (%)	F-Score (%)
50 + 100	95.3	94	96.5	95.2
50 + 100 + 150	96.5	94.67	98.36	96.49
50 + 100 + 150 + 200	93.6	91.37	95.29	93.29
50 + 100 + 150 + 200 + 250	90.8	86.84	94.48	90.49
50 + 100 + 150 + 200 + 250 + 300	88.4	87.35	87.53	87.44

It is also shown in Table 10 that the combination of the fundamental wave with the second and third harmonics yields the best effect in this experiment. However, this combination method may not be universally optimal across various audio frequencies due to the differential destruction of high-order harmonic signals. In the binary-class scenario, the accuracy of the 1D-CNN model saw a significant improvement, rising from 87.76 to 96.5%, when progressing from experiments with a single feature component to those incorporating multiple feature components. According to the experimental results, the network is able to learn more specific information from the combined input that contains the ENF fundamental and its harmonics. Although employing ENF harmonic component independently as a single-channel input achieved commendable detection accuracy, the synthesized input enriches the feature set, thereby further elevating precision and augmenting the model’s overall efficacy. The experiments in this chapter demonstrate the applicability and robustness of the 1D-CNN model for tampering detection scenarios as well as the advantage of the combination of multi-feature components.

## Conclusions

This study presents a novel framework for audio tampering identification based on blind forensics technology utilizing ENF and deep learning. Moreover, it proposes an input method that combines the fundamental and harmonic features of the ENF signal. The proposed framework, termed Audio Tampering Detection (ATD), simplifies the process of audio forensics and identification by eliminating the need for manual threshold setting in signal classification. Instead, it utilizes ENF signal features extracted from the audio data itself as input for the model. Based on the differentiation of audio tampering types, the experiments evaluated the performance of the model respectively in binary-class and four-class tampering detection, where the ATD framework achieves an accuracy of over 93% in the four-class scenario and over 96% in the binary-class scenario. These experimental findings indicate that the proposed ATD framework can accurately identify the type of audio tampering and enhance the robustness of the classification model. In future studies, emphasis will be put on the application of blind forensics with ENF technology in video data, thereby expanding the scope of our research beyond audio analysis.

## Data Availability

Data sets generated during the current study are available from the corresponding author on reasonable request.
